# Health and Demographic Surveillance System in the Western and Coastal Areas of Kenya: An Infrastructure for Epidemiologic Studies in Africa

**DOI:** 10.2188/jea.JE20110078

**Published:** 2012-05-05

**Authors:** Satoshi Kaneko, James K’opiyo, Ibrahim Kiche, Sheru Wanyua, Kensuke Goto, Junichi Tanaka, Mwatasa Changoma, Morris Ndemwa, Osuke Komazawa, Mohamed Karama, Kazuhiko Moji, Masaaki Shimada

**Affiliations:** 1 Department of EcoEpidemiology, Institute of Tropical Medicine, Nagasaki University, Nagasaki, Japan; 2 Centre of Public Health Research, Kenya Medical Research Institute (KEMRI), Nairobi, Kenya; 3 NUITM-KEMRI Project, Kenya; 4 Research Institute for Humanity and Nature, Kyoto, Japan

**Keywords:** health and demographic surveillance system, Kenya, cohort profile, INDEPTH Network

## Abstract

**Background:**

The Health and Demographic Surveillance System (HDSS) is a longitudinal data collection process that systematically and continuously monitors population dynamics for a specified population in a geographically defined area that lacks an effective system for registering demographic information and vital events.

**Methods:**

HDSS programs have been run in 2 regions in Kenya: in Mbita district in Nyanza province and Kwale district in Coast Province. The 2 areas have different disease burdens and cultures. Vital events were obtained by using personal digital assistants and global positioning system devices. Additional health-related surveys have been conducted bimonthly using various PDA-assisted survey software.

**Results:**

The Mbita HDSS covers 55 929 individuals, and the Kwale HDSS covers 42 585 individuals. In the Mbita HDSS, the life expectancy was 61.0 years for females and 57.5 years for males. Under-5 mortality was 91.5 per 1000 live births, and infant mortality was 47.0 per 1000 live births. The total fertility rate was 3.7 per woman. Data from the Kwale HDSS were not available because it has been running for less than 1 year at the time of this report.

**Conclusions:**

Our HDSS programs are based on a computer-assisted survey system that provides a rapid and flexible data collection platform in areas that lack an effective basic resident registration system. Although the HDSS areas are not representative of the entire country, they provide a base for several epidemiologic and social study programs, and for practical community support programs that seek to improve the health of the people in these areas.

## INTRODUCTION

**Figure 1.**
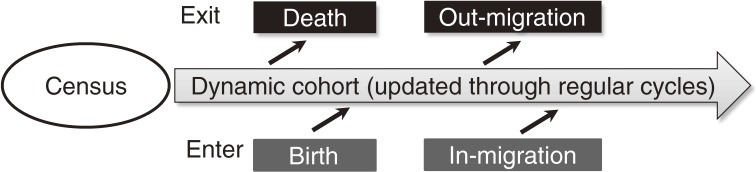
Basic structure of Health and Demographic Surveillance System (HDSS). During the first phase, a census is conducted to register all residents in the HDSS area. Then, routine follow-up rounds update information on people exiting and entering the dynamic cohort of the population covered.

A Health and Demographic Surveillance System (HDSS) is a longitudinal data collection process that systematically and continuously monitors population dynamics in a specified population in a geographically defined area that lacks an effective system for registering demographic information and vital events.^1^^–^^3^ The simplest HDSS consists of prospective data collection of vital events such as births, deaths, and migrations among the population, with periodic updates made via visits to all households in the defined area (Figure 1). A more advanced HDSS adds various surveys during follow-up periods, to assess other variables (such as health-related factors and socioeconomic factors), to investigate risks of diseases or health conditions, or to identify high-risk groups among communities in the area. The data collected by the HDSS can be used not only for descriptive and analytic epidemiologic studies, but also for community-based interventional studies like a cluster-randomized trial,^4^^,^^5^ which allocates treatment arms randomly to groups of individuals referred to as “cluster,” eg, a community, village, or area in the HDSS area. The HDSS can be used as a base or infrastructure to test a new methodology of disease control in an area with no civil registration system.^6^^,^^7^

The concept of the HDSS is not new.^8^ Since the early 20th century, a study concept existed in which a community would be prospectively and systematically observed to collect public health, epidemiologic, and demographic information.^8^^,^^9^ This type of study was called a population laboratory or population observatory at that time, because it observed a whole community or population for the purpose of research.^10^ In 1997, Garenne et al renamed this type of study a prospective community study. Initially, the term demographic surveillance system (DSS) was used to refer to a system for managing demographic data in a prospective community study program.^8^ In 1998, some prospective community study groups gathered to share information on DSSs and organized a new association.^3^ This new alliance of DSSs was organized and named the INDEPTH Network (International Network for the Demographic Evaluation of Populations and Their Health in Developing Countries). At that time, the term DSS replaced prospective community study. In 2009, INDEPTH added the word “Health” before DSS, and the term became Health and Demographic Surveillance System (HDSS). However, the main purpose of the HDSS remained the same, that is, observation of population dynamics in a specific geographic area to support epidemiologic and interventional studies.^11^ The value of the HDSS as a stable and reliable source of information has been increasing with regard to health and demographic data from areas and regions that lack data collection systems for vital statistics.^2^^,^^3^^,^^12^^,^^13^

The Institute of Tropical Medicine at Nagasaki University is a research institute for tropical medicine and public health in low- and middle-income countries in Asia and Africa. It launched a new program in Kenya in 2005 that uses an HDSS as an infrastructure. The aim of this program was to conduct studies of infectious and parasitic diseases, other health conditions, and socioeconomic and environmental factors.^11^^,^^14^ As of 2011, we have set up and implemented 2 HDSS programs in the Western and coastal areas of Kenya, which are areas that have different disease burdens and cultures. In this article, we describe the profile of our HDSS programs in Kenya and share some basic findings from the HDSS dataset.

## METHODS

### Study area

**Figure 2.**
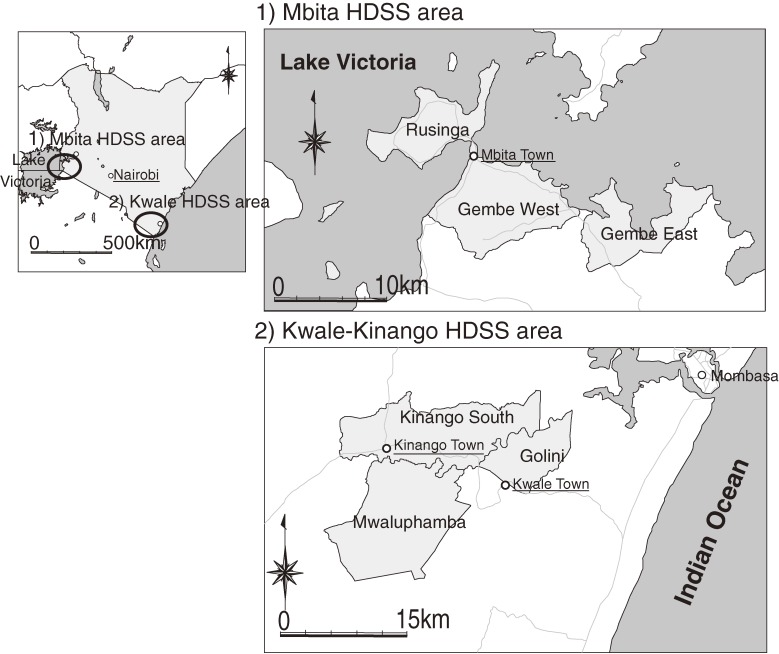
Health and Demographic Surveillance Area: the NUITM-KEMRI project. The NUITM-KEMRI project was launched in 2005 to enhance research in tropical medicine and train young Kenyan and Japanese scientists. NUITM, Nagasaki University Institute of Tropical Medicine; KEMRI, Kenya Medical Research Institute.

Our HDSS programs are run in 2 regions of Kenya, ie, the Mbita district in Nyanza province and the Kwale district in Coast Province (Figure 2). In Mbita, the HDSS program (hereafter “Mbita HDSS”) follows 11 182 households and 55 929 inhabitants as of 1 July 2011 in an area of 163.28 km^2^ between latitudes 0°21′S and 0°32′S and longitudes 34°04′ and 34°24′, which includes Rusinga West, Rusinga East, Gembe West, and Gembe East. In Kwale, the HDSS program (hereafter “Kwale HDSS”) covers 7617 households and 42 585 inhabitants as of 1 July 2011 in the Kinango South and Mwaluphamba locations of the Kwale district in an area of 384.9 km^2^, which includes 3 locations between latitudes 4°17′S and 4°5′S and longitudes 39°15′ and 39°29′: Mwaluphamba, Kinango South, and Golini. The Golini location (58.3 km^2^), which includes the eastern side of the Kwale-Kinango HDSS, was added in March 2011 to expand the area of coverage.

We selected these 2 HDSS areas with different cultural and environmental backgrounds to compare disease patterns and factors affecting health status. Additionally, we considered the following characteristics in the selection process: (1) suitability population density for an HDSS, (2) existence of currently targeted diseases by our researchers (malaria, schistosomiasis, filariasis, human immunodeficiency virus [HIV], tuberculosis, and diarrhea, (3) absence of other organizations operating in the study area, and (4) practicality of establishing a field station. After considering the above characteristics and activities to date by the researchers from Nagasaki University, 2 local areas in Mbita and Kwale were selected as HDSS sites. Malaria, schistosomiasis, filariasis, HIV, and tuberculosis are endemic in the region around the Mbita and Kwale HDSS areas.^15^ Also, the areas had different cultural backgrounds, including lifestyle, environment, and religion. In the Mbita HDSS area, Christianity is the main religion, and people live on fishing in Lake Victoria and peasant farming. In contrast, in the Kwale HDSS area, the main religion is Islam, and the people are peasant farmers who rely on subsistence agriculture for food.

The field station in Mbita is located in the International Centre for Insect Physiology and Ecology (ICIPE) research compound. In Kwale, the field station is set up in the community resource centre of Kwale district. Both stations have electricity with a backup generator and internet connection and are equipped with a data server, personal computers, scanner, printers, and motorcycles to manage the HDSS.

All the individuals recognized by our field interviewers during the baseline census and follow-up rounds (described below) are registered under the HDSS programs, except for visitors to the areas.

### Data collection methods

#### Baseline census

Using software for personal digital assistants (PDAs)—ie, a small device resembling a computer that uses a pencil-like stick (stylus) instead of a keyboard to enter data—and a global positioning system (GPS) that we developed for this project, all households and all members in the HDSS areas were registered by our local field interviewers. In the Mbita HDSS, we started the baseline census between August and December of 2006 without GPS information; however, we re-registered the population, including the residents who had temporarily migrated out of the area, using the GPS between October and December 2008. In the Kwale HDSS, the baseline census was conducted between July 2010 and December 2010. From April 2011, the Kwale HDSS was expanded to the Golini location, a location adjacent to the original area, and the baseline survey finished in July 2011.

#### Routine follow-up at fixed intervals

During routine follow-up rounds, vital events such as in-migration, out-migration, pregnancy, death, and deliveries and numbers of newborns are recorded using the PDA. Data are updated at 1- to 2-month intervals. Because all records have already been stored on a Structured Query Language (SQL) database in the PDA, the field interviewers only register or update the events according to the PDA program instructions. The HDSS program updates the registered information on households at least once a year. When new families or residents migrate into the HDSS area or babies are born, they are registered in the system.

#### Extra-survey program (pop-up program)

During every follow-up round, we add different types of questions to the routine HDSS event update; the new questions appear (pop up) on the PDA as part of the routine updating program (thus we call it a “pop-up program”). For example, we added surveys on water sources, school attendance, bed-net usage, health-seeking behavior, immunization history, breastfeeding and child care, and dental hygiene, among others. The pop-up program also provides a sampling function for random sampling surveys, such as sampling 10% of all residents in the HDSS area or using selected surveys only for children younger than 5 years.

#### Verbal autopsy

The verbal autopsy (VA) is a process that attempts to determine cause-of-death in areas with incomplete or no vital registration systems. In many less-developed areas, most deaths occur at home without a diagnosis, which makes it difficult for health planning, priority setting, monitoring, and evaluation.^16^^,^^17^ Field interviewer managers, who are the first level of supervisors, visit houses where deaths were registered by field interviewers, and conduct a VA using VA forms. After the VA forms are completed, they are given to physicians or clinical officers who read the history of illness and provide a cause of death.

### System for data collection

#### System development for PDA devices and GPS

As part of the HDSS registration system, we developed a software program for the PDA that enables us to conduct paperless registration and follow-up for households and individuals in the HDSS areas. By using a paperless system, we save paper normally used for registration and follow-up data, do not need space for saving the filled-in forms, reduce personnel costs to enter data from the registration and follow-up forms, and save time entering and analyzing data. Furthermore, the PDA program can assist surveys and data entry for field interviewers by giving instruction and on-site validation checking of entered data. It also generates house and individual identification numbers (IDs) for new registrants, so that no person needs to assign and re-register IDs, as would be required in a paper-based registration system. Our PDA system was developed using the Microsoft .Net framework and SQL server compact 3.5. The database consists of 15 tables: 10 tables for registration and follow-up and 5 tables for administration and management of the PDA program.

#### Grid geographical address system

**Figure 3.**
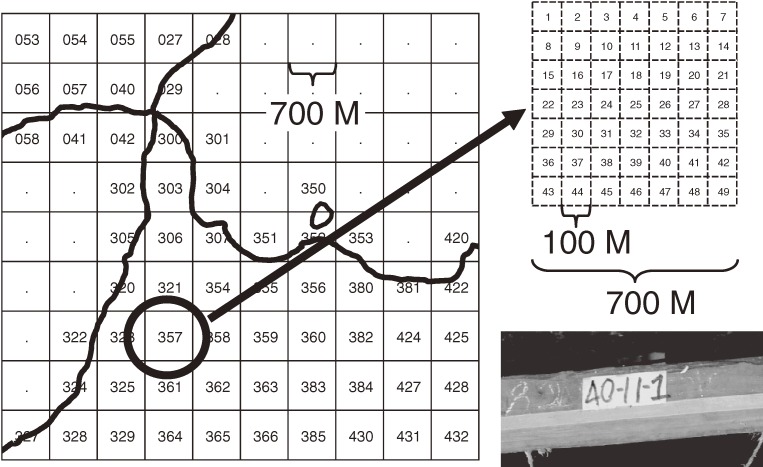
Grid geographical address system (GGAS). Each grid is a square (700 m^2^) that is divided into 49 subgrids (eg, left square). The subgrids are numbered from 1 to 49, from the left upper edge to the right lower edge. Each residential structure is numbered using the grid, subgrid, and sequence number within the grid-subgrid area, which is written on the door of the structure. The geographical position of each house ID is automatically recorded in the database at registration, using the longitude and latitude obtained by the GPS receiver.

In both HDSS areas, not all houses have addresses to show dwelling places, which means we need a system to identify house structures in our HDSS program. To provide a house identification that enables us to recognize geographic areas and locations easily, we developed a grid geographical address system that uses longitude and latitude given by the GPS mounted on the PDA. As shown in Figure 3, the HDSS areas were divided using uniquely numbered squares with 700-meter sides. Each 700-m^2^ grid is further subdivided into 49-square grids (subgrids) with sides measuring 100 meters. Each subgrid has a number from 1 to 49. Furthermore, we provide a sequential number to a house structure within the same subgrid. As a result, each house structure has an ID number, eg, 357-32-12, that has its geographical position automatically recorded in the database at registration using the longitude and latitude obtained by the GPS receiver.

### Quality control and quality assurance

An essential issue in a data collection system like the HDSS, which deploys field workers in wide areas, is control of data quality. We have implemented several systems to prevent errors and misconduct by field interviewers such as intentionally or unintentionally skipping questions and entering or updating data without actually visiting houses. To avoid such behaviors, the PDA program uses a system that checks for missing and incompatible data on the spot, during the interview. In addition, it includes a program that calculates the distance between the place where the field interviewer is standing and the location where the house is registered when the field interviewer starts updating data. This program prevents the data updating program from starting if the field interviewer is standing more than 20 meters away from the target house. We are also able to monitor working hours of the field interviewer using times recorded on each record in the PDA database when a field interviewer updates any data. We also validate data collected by the field interviewer by random spot checking, which is done by second-level and third-level managers.

### Organizing HDSS management

To run our HDSS programs, we assign a field manager for each HDSS site to manage the overall program in the area. Under the manager, 4 field-interviewer managers (FIMs) are deployed to manage routine data collection conducted by field interviewers (FIs). During recruitment of FIs and FIMs, applicants submitted the application form for each position to us with copies of their national ID card, their grade transcript for the Kenya Certificate of Secondary Education (KCSE), which is the annual nationwide examination taken at the completion of secondary education, and other certificates and diplomas. After applicant screening, those who had a KCSE grade of C^−^ or higher (for FIs) plus additional diplomas and some basic community experience (for FIMs) were selected. Then, a 90-minute examination was given. For those with a higher exam result, an interview exam was given as part of further selection. After selection, training for the HDSS job was given to FIMs for only 3 weeks (1 week for lectures, 1 week for basic PDA training, and 1 week for field training). Then, trained FIMs gave the same training to FIs, under our supervision. We recruited local individuals for FI and FIM positions because they understand the local language and communities. Currently, 16 FIs in Mbita and 10 FIs in Kwale are working in their assigned areas to register and update information on vital events and to complete additional surveys provided by the PDA pop-up program. FIMs visit areas to replace PDA batteries and collect data from the PDA twice a week. Collected data are transferred to an SQL database (MySQL 5.0) in the local data server to monitor and evaluate the progress of data collection for the HDSS program in each round at local level.

### Data transfer from the field station

After transferring data from the PDA to the local server in the field station, PDA data are sent to a Nairobi server via the Internet, using a secure channel to manage both HDSS programs. Internet service in Kenya is not always stable. To address this instability in the internet networks, we accumulate daily datasets and send any remaining datasets when the internet becomes available. To avoid traffic congestion on the internet during the day, which might result in transfer errors, we send data at night, when internet traffic is relatively light. The transferred PDA data is automatically stored in the SQL database in the server; then auto-analysis programs monitor the progress of the HDSS follow-up rounds, working hours of field interviewers, and new deaths for VA, among other functions. For data management, STATA version 10 (Stata Corporation, TX, USA) is used after loading data directly from the SQL database by using the STATA ODBC command until the auto-analyses for data management.

### Ethical considerations

The head of the household must give informed consent for HDSS registration and follow-up. To maintain a good relationship between our HDSS program and communities, we have routine meetings for community sensitization, which improve consent rates for participation in the registration. For households that reject registration at the first visit by an FIM, our field manager and village elders visit the household to explain our program and persuade them to join our HDSS program. Most such cases are households that have recently migrated into the HDSS area, and they usually agree to be registered and followed after they understand the program. The protocols for the Mbita HDSS and Kwale HDSS were reviewed and approved by the Ethical Review Committee of KEMRI (KEMRI SSC No. 1088) and the Institutional Review Board of the Institute of Tropical Medicine, Nagasaki University (IRB # 06060604).

## RESULTS

**Figure 4.**
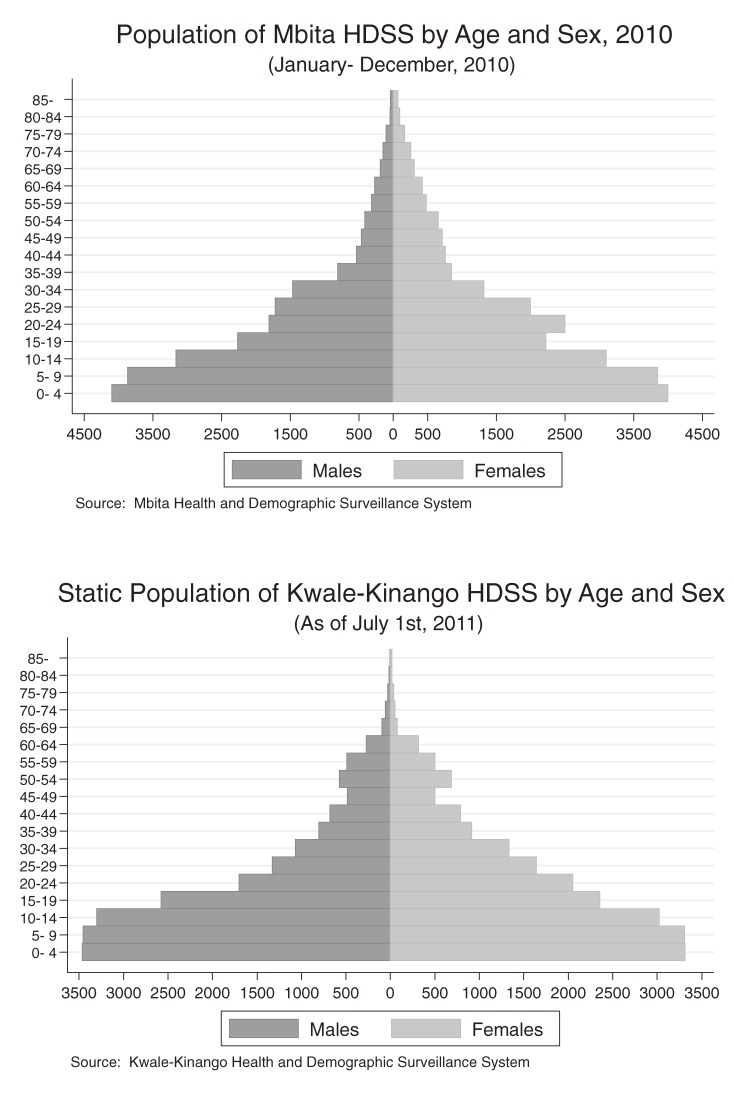
Population pyramids of the Mbita HDSS and Kwale HDSS. The population of the Mbita HDSS (above) is calculated using person-years observed between January 2010 and December 2010, excluding individuals who stayed less than 2 months in the HDSS area per year. The population of the Kwale HDSS (below) is calculated using the number of residents as of July 2011, because this HDSS has been observed for less than 1 year.

Only basic statistics are shown in this paper because much of the data are currently being analyzed or are still being organized for analysis. Figure 4 shows the population pyramids of the 2 HDSS areas. Populations in the HDSS programs are usually calculated using person-years, which are based on the person-time contributed by each registered resident. In calculating person-time in our program, we defined residents as those who stayed in the HDSS area 60 days (2 months) a year or longer; thus, registered individuals who stayed less than 60 days a year were removed for the calculation of total person-years. In the Mbita HDSS, the person-years observed from 1 January 2010 to 31 December 2010 were calculated. In the Mbita HDSS there were 45 493.4 person-years from 53 437 residents in the year 2010. In contrast, in the Kwale HDSS, which is a static population, 42 585 residents were calculated as of 1 July 2011, because the Kwale HDSS has been in operation for less than 1 year at the time of this report.

Table 1 shows demographic statistics from the Mbita HDSS program; most rates were calculated with the person-year denominator. In 2010, the Mbita HDSS recorded 1351 live births and 416 deaths. From the HDSS database, we made a life table according to the INDEPTH network.^1^ Life expectancy in this area in 2010 was 61.0 years for females and 57.5 years for males. Under-5 mortality was 98.0 per 1000 live births for girls and 84.9 for boys (91.5 per 1000 live births in total). Infant mortality was 55.4 for girls and 38.4 for boys per 1000 live births and 47.0 per 1000 live births in total in 2010. In Mbita, the total fertility rate, ie, the average number of children that would be born per woman if all women lived to the end of their childbearing years^18^ (defined as age 49 years in this research) was 3.7.

**Table 1.** Basic demographic data collected in the Mbita Health and Demographic Surveillance System in 2010

Index	Value	Unit
Crude birth rate	29.7	per 1000 person years
Total fertility rate	3.7	per woman
Crude death rate	9.1	per 1000 person years
Neonatal mortality rate	14.1	per 1000 live birth
Post-neonatal mortality rate	33.0	per 1000 live birth
Infant mortality	47.0	per 1000 live birth
Under-5 mortality	91.5	per 1000 live birth
Crude rate of natural increase	2.1	%
In-migration rate	64.1	per 1000 person years
Out-migration rate	86.2	per 1000 person years
Life expectancy at birth (females)	61.0	years
Life expectancy at birth (males)	57.5	years

Mortality rates by age group in 2010 are shown in Table 2. Infant mortality for girls (58.3/1000 person-years) was slightly higher than for boys (39.8/1000 person-years). Mortality rates for adults aged 20 to 29 years, which is child-bearing age in this area, were higher among women than among men. We counted 31 deaths in 4500 person-years among women aged 20 to 29 years as compared with 13 deaths in 3523.2 person-years among men of the same age. From age 30 years, mortality rates among men were higher than those among women in this area.

**Table 2.** Mortality rates by age group and sex in Mbita HDSS area, 2010

Age group	Females			Males			Total		
		
	Deaths	Person- years	Mortality rate per 1000 py	Deaths	Person- years	Mortality rate per 1000 py	Deaths	Person- years	Mortality rate per 1000 py
0	41	703.1	58.3	28	703.4	39.8	69	1406.5	49.1
1–4	38	3296.8	11.5	42	3394.7	12.4	80	6691.4	12.0
5–9	8	3849.9	2.1	12	3867.2	3.1	20	7717.0	2.6
10–14	2	3101.7	0.6	0	3158.8	0.0	2	6260.5	0.3
15–19	3	2226.2	1.3	4	2266.9	1.8	7	4493.2	1.6
20–24	11	2501.9	4.4	5	1808.1	2.8	16	4310.1	3.7
25–29	20	1998.9	10.0	8	1715.2	4.7	28	3714.1	7.5
30–34	8	1325.4	6.0	24	1462.0	16.4	32	2787.4	11.5
35–39	7	848.8	8.2	10	809.0	12.4	17	1657.8	10.3
40–44	6	758.0	7.9	11	535.9	20.5	17	1293.9	13.1
45–49	9	712.7	12.6	9	465.0	19.4	18	1177.8	15.3
50–54	6	654.4	9.2	10	415.8	24.1	16	1070.2	15.0
55–59	11	485.8	22.6	7	315.3	22.2	18	801.1	22.5
60–64	9	425.0	21.2	5	272.8	18.3	14	697.8	20.1
65–69	5	309.5	16.2	6	189.0	31.7	11	498.5	22.1
70–74	9	255.0	35.3	8	148.9	53.7	17	403.9	42.1
75–79	5	165.6	30.2	6	101.5	59.1	11	267.1	41.2
80–84	8	95.9	83.4	2	43.9	45.5	10	139.8	71.5
85–	10	69.9	143.0	3	36.0	83.3	13	106.0	122.7

Total	216	23 784.6	9.1	200	21 709.4	9.2	416	45 494.0	9.1

Abbreviation: py, person-years.

Table 3 shows house structures and house properties that were registered in the Mbita HDSS and Kwale HDSS after updating data and basic registration for new households. These data can be used as variables of socioeconomic status in epidemiologic studies. In Mbita, as briefly summarized, most households (89%) used a lake as their main source of drinking water, and the proportion of households with toilets was lower than 40%. However, in Kwale, piped water was used as the main source of drinking water in 51.6% of households, although the proportion of households with toilets was almost the same as that for Mbita.

**Table 3.** House structure as identified in the Mbita and Kwale Health and Demographic Surveillance Systems (HDSSs)

		Mbita HDSS		Kwale HDSS	
			
		Freq.	%	Freq.	%
**1) Number of rooms**					
	1	2516	22.5	1040	13.7
	2	4727	42.3	3061	40.2
	3	2831	25.3	2157	28.3
	4	606	5.4	1023	13.4
	5	185	1.7	143	1.9
	More Than 5	152	1.4	171	2.2
	Missing	165	1.5	22	0.3
**2) Structure of wall**					
	Stone	354	3.2	62	0.8
	Wood and Mud	6948	62.1	6537	85.8
	Brick and/or Block	999	8.9	479	6.3
	Grass or Reeds	5	0.0	7	0.1
	Iron Sheets	1066	9.5	15	0.2
	Mud and Cement	1620	14.5	492	6.5
	Other	1	0.0	2	0.0
	Tin	3	0.0	1	0.0
	Wood and Timber	21	0.2	0	0.0
	Missing	165	1.5	22	0.3
**3) Structure of floor**					
	Earth, Dung or Sand	7515	67.2	6743	88.5
	Carpet	6	0.1	0	0.0
	Cement	3482	31.1	767	10.1
	Ceramic Tiles	1	0.0	2	0.0
	Palm or Bamboo	1	0.0	0	0.0
	Tiles or Linoleum	7	0.1	8	0.1
	Wood Planks	5	0.0	69	0.9
	Other	0	0.0	6	0.1
	Missing	165	1.5	22	0.3
**4) Structure of roof**					
	Asbestos Sheet	5	0.0	3	0.0
	*Makuti* (Palm leaves)	1	0.0	5404	71.0
	Tiles	2	0.0	2	0.0
	Concrete	26	0.2	2	0.0
	Grass or Thatch	1010	9.0	250	3.3
	Iron Sheets (*Mabati*)	9969	89.2	1929	25.3
	Tin or Metal Sheet	4	0.0	3	0.0
	Other			2	0.0
	Missing	165	1.5	22	0.3
**5) Main source of drinking water**					
	Borehole	118	1.1	7	0.1
	Lake	9949	89.0	12	0.2
	Piped	124	1.1	3927	51.6
	Covered Well	6	0.1	20	0.3
	Open Well	1	0.0	1131	14.9
	Pond or Dam	249	2.2	1245	16.4
	Rainwater with Tanks	13	0.1	36	0.5
	Spring	54	0.5	273	3.6
	Stream or River	498	4.5	941	12.4
	Missing	165	1.5	22	0.3
	Other	5	0.0	3	0.0
**6) Manner of disposing of garbage**					
	Garbage	4078	36.5	5773	75.8
	Abandoned House	92	0.8	4	0.1
	Burning	3876	34.7	130	1.7
	Dumping Pit	2605	23.3	523	6.9
	Lake	10	0.1	1	0.0
	River or Stream	165	1.5	61	0.8
	Road	76	0.7	0	0.0
	Other	115	1.0	1103	14.5
	Missing	165	1.5	22	0.3
**7) Toilet**					
	No Toilet	7349	65.7	4523	59.4
	Flush Toilet	5	0.0	22	0.3
	Pit latrine	3560	31.8	3023	39.7
	Ventilated pit latrine	103	0.9	27	0.4
	Missing	165	1.5	22	0.3
**8) Lighting**					
	Electricity	212	1.9	177	2.3
	Lantern	3539	31.7	137	1.8
	Pressure Lamp	77	0.7	51	0.7
	Tin Lamps	7171	64.1	7207	94.6
	Other	18	0.2	23	0.3
	Missing	165	1.5	22	0.3
**9) Heat source for cooking**					
	Charcoal	1535	13.7	275	3.6
	Electricity	5	0.0	20	0.3
	Fire Wood	9393	84.0	7234	95.0
	Gas	30	0.3	6	0.1
	Solar	7	0.1	38	0.5
	Other	47	0.4	22	0.3
	Missing	165	1.5	22	0.3
**10) Property possessed**					
	Car	56	0.5	28	0.4
	Fridge	47	0.4	15	0.2
	TV	826	7.4	235	3.1
	Iron box	2279	20.4	464	6.1
	Cellular phone	4596	41.1	1806	23.7
	Motorcycle	318	2.8	110	1.4
	Radio	8732	78.1	2913	38.2
	Bicycle	2370	21.2	1753	23.0
	Sofa	7615	68.1	437	5.7
	Cross table	10 601	94.8	3407	44.7
	Bed	10 661	95.3	6706	88.0
	Livestock	4759	42.6	4512	59.2
	Poultry	7262	64.9	6465	84.9
**11) Owner of land**					
	Family Land	4196	37.5	5465	71.8
	Government Land	130	1.2	40	0.5
	Leased Land	1965	17.6	84	1.1
	Own Land	4501	40.3	1902	25.0
	Squatter	225	2.0	104	1.4
	Missing	165	1.5	22	0.3

**Total**		11 182	100.0	7617	100.0

## DISCUSSION

The presence of an HDSS in an area with no proper residential and vital registration system provides basic and statistical infrastructure for demographics and vital statistics without any additional investment by researchers, local officials, or communities. As a result, in addition to our HDSS program, we have several research programs and grassroots projects that support communities in the HDSS area, as shown in the Appendix.^19^^,^^20^ Although HDSS programs have advantages, most HDSS programs in other areas require additional time for entering data from paper-based record forms into databases and for data cleansing. This process can take several months and can greatly prolong the period between data collection and analysis. Our paperless HDSS program, which uses a PDA, GPS, and the internet, enables us to analyze data on the day when data are collected. Moreover, this system provides on-the-spot logical checks of data entered by field interviewers, which also saves time and reduces human error and the need for additional resources. It also permits checking of when and where data were updated for each household, thereby ensuring data quality.

Among HDSS centers participating in the INDEPTH Network, one goal is to share information more widely to provide common data to analyze global health problems.^21^^,^^22^ The 2 HDSS programs detailed here will be able to provide information to other centers and contribute information to the international community. Furthermore, our HDSS program has additional, educational value for young scientists. It provides good training and research opportunities for undergraduate and graduate students. Moreover, we have developed several technological improvements to better manage the HDSS system and data collection.

Another advantage is the ability to use the HDSS system in other areas. Our system, originally developed for the Mbita HDSS program, was easily transplanted to the Kwale HDSS. Regarding areas outside Kenya, in 2010 our system was transplanted to the Lahanam HDSS program in the Lao People’s Democratic Republic, where it is operated collaboratively by the National Institute of Public Health, Laos and the Research Institute for Human and Nature, Kyoto, Japan after being translated from English to Laotian. The Laotian HDSS program covers about 7500 inhabitants, but plans are underway to expand to surrounding areas.

Although an HDSS can yield a variety of demographic and health-related data, there has been some criticism of HDSS data for not being representative of the population and the area to which the data are extrapolated.^23^ This loss of representativeness or generalizability is unavoidable and intrinsic to HDSS programs because these programs change the communities and areas by providing benefits from the HDSS program itself or from programs secondarily introduced to the communities in the HDSS area. Such programs make the areas different from areas without HDSS programs. For example, using analysis and data provided by the HDSS program, a district government can plan a community health strategy in an area.^24^^,^^25^ Also, research teams can design a more detailed plan for health research because the basic statistical data are provided by the HDSS program, which improves community health as an outcome of research.^23^^,^^26^^–^^28^ The potential loss of generalizability in HDSS programs has been discussed for some time. However, we believe that the numerous advantages of the current program outweigh the disadvantages because there is a lack of reliable information in the developing world. These advantages include the benefits of having precise, timely data and opportunities to systematically evaluate public health interventions, as well as the usefulness of HDSS data for understanding areas of the developing world where no information is available.^21^ Additionally, with regard to the Millennium Development Goals, the research agenda is shifting toward large-scale, multicenter trials and accelerated efforts at disease control.^22^^,^^29^

In summary, our HDSS programs provide a rapid and flexible data collection platform in less-developed areas that lack an effective basic resident registration system.^22^ This enables us to conduct several epidemiologic and social study programs and provide practical programs for community support to sustain healthy lives in such areas.

**Appendix.** Programs and projects funded and running in the HDSS areas

1) Research activities funded			Period
	a.	Bed-net project for prevention of malaria in Mbita HDSS area, funded by Sumitomo Chemical Co., Ltd.	2009–2012
	b.	Child sub-cohort program in Mbita and Kwale HDSS area, funded by Grant-in-Aid for Scientific Research (B) (KAKENHI) from the Ministry of Education, Culture, Sports, Science and Technology of Japan (MEXT)	2010–2013
	c.	Research on neglected tropical diseases, funded by Special Coordination Funds for Promoting Science and Technology (SCF) from MEXT	2009–2011
	d.	Parasitological research, funded by a government grant for tropical disease research and clinico-epidemiologic research at Nagasaki University	2010–2016
	e.	Malaria elimination project, funded by a government grant for tropical disease research and clinico-epidemiologic research at Nagasaki University	2010–2016
	f.	Dental health project, funded by Nagasaki University	2010–2016

2) Community support (grassroots) projects funded			
	a.	JICA grassroots technical cooperation program, titled “Enhancement of health service delivery and capacity building of health personnel for the poor through community participation in Western Kenya” in the Mbita HDSS area	2009–2011
	b.	Grant assistance for grassroots projects (GGP,) provided by the Government of Japan through the Embassy of Japan in Kenya in Mbita	2010
